# Editorial: Regulators of radiosensitivity in colorectal cancer

**DOI:** 10.3389/fonc.2022.1094238

**Published:** 2022-11-29

**Authors:** Kumar Bishnupuri, Shuyu Zhang

**Affiliations:** ^1^ Washington University School of Medicine, St. Louis, MO, United States; ^2^ The Second Affiliated Hospital of Chengdu Medical College, China National Nuclear Corporation 416 Hospital, Chengdu, China; ^3^ Laboratory of Radiation Medicine, West China Second University Hospital, Sichuan University, Chengdu, China; ^4^ Laboratory of Radiation Medicine, West China School of Basic Medical Sciences & Forensic Medicine, Sichuan University, Chengdu, China; ^5^ National Health Commission (NHC) Key Laboratory of Nuclear Technology Medical Transformation (Mianyang Central Hospital), Mianyang, China

**Keywords:** irradiation, radiosusceptibility, radiotherapy, resistance, survival

Ionizing radiation has been used for the treatment of cancer since 1896, shortly after Roentgen’s discovery of X-rays (1). The term ‘radiosensitivity’ is one of the most extensively used words in oncology. It was initially used by French as “radiosensibilité” and by Germans as “Strahlenempfindlichkeit” in 1907 (2). The French “radiosensibilité” was likely originated from “radioactivité”, proposed by Curie to replace the term “hyper-phosphorescence” initially chosen by Becquerel following his discovery of natural radioactivity in 1896. While the term “radiosensitivity” was used by the pioneers to describe only the predisposition to the radiation-induced adverse tissue reactions related to cell death, a confusion emerged in the literature from the 1930s when English became the official language during the first International Congresses of Radiology, and the term “radiosensitivity” was indifferently used to describe the toxic, cancerous, or aging effect of irradiation (3). Accordingly, the mechanism of radiosensitivity, notably observed after radiotherapy appeared to be different than those linked to predisposition to radiation-induced cancer, commonly termed as radiosusceptibility. The separate use of the terms ‘radiosensitivity’ and ‘radiosusceptibility’ has been suggested to describe variability in the risk of, respectively, adverse tissue reactions (deterministic effect) following radiotherapy and radiation-induced cancer (stochastic effect).

Cancer cells respond to irradiation in a heterogeneous manner depending on their intrinsic properties for regulating their DNA damage repairs, mitochondrial functions, distribution of cells in different phases of the cell cycle, and tendency to undergo apoptosis. Furthermore, extrinsic environment such as the degree of hypoxia within the tumor population is also an important factor for regulating response of cancer cells to radiotherapy (RT). In addition, the degree of radiosensitivity of cancer cells depends on the cell heterogeneity within the tumor mass that includes cancer-initiating cells or cancer stem cells. In recent years, researchers in oncology identified potential genes, miRNAs, RNA-binding proteins, and stem cells-associated factors regulating the radiosensitivity of cancer cells, hence the overall effectiveness of radiotherapy.

Radiotherapy is a standard treatment for solid tumors and over 50% of patients with cancer receive irradiation as therapy. While radiotherapy has significantly improved the overall survival and quality of life of cancer patients, its efficacy has still been markedly limited by radioresistance in a significant number of cancer patients, resulting in failure of the radiotherapeutic control of the disease. Radioresistance leads to cancer recurrence, metastasis, and poor prognosis of cancer patients, hence constitutes a substantial barrier to the success in cancer management. Accordingly, understanding the molecular mechanisms that underlie both intrinsic and acquired radioresistance to cancer cells is extremely important to develop effective treatments to overcome the malignancy permanently.

One of the major problems linked to the current is the fact that they are optimized for decreasing tumor in bulk by acting mainly on the non-stem cancer cells while leaving cancer stems cells (CSCs) untouched. Moreover, these cancer cells have been demonstrated to be highly resistant to cancer treatments, owing to different intrinsic characteristics typical to these cells. CSCs have an important role in acquired resistance too, that develops because of cancer treatments and has been associated with different biological processes. Asymmetric division of cancer treatment-resistant CSCs contribute to cell heterogeneity within the tumor and increase the abundance of the CSC population (4, 5). In addition, the tumor microenvironment plays an important role in the acquisition of radioresistance. Some immune cells, cancer-associated fibroblasts (CAFs) and even adipocytes contribute to the development of a resistant phenotype through the production of several soluble factors, such as cytokines, chemokines, and metabolites (6), which stimulate molecular mechanisms involved in the development of a radioresistant phenotype.

Overall, radiosensitivity of cancer cells, in a broader spectrum, provides a fundamental basis of cancer treatment management. This Research Topic of the ‘Frontiers in Oncology’ journal is focused on defining/identifying biological factors both within the tumor and in the surrounding microenvironment regulating cell cycle, oxidative stress, hypoxia, apoptosis, DNA damage/repair, mitochondrial function, inflammation, and stem cell survival with a broader aim to expand our knowledge of cancer cell radiosensitivity, and its implication in improving cancer patient outcomes.

## Author contributions

KB: Conceptualization and writing; SZ: Writing. All authors contributed to the article and approved the submitted version.

## Conflict of interest

The authors declare that the research was conducted in the absence of any commercial or financial relationships that could be construed as a potential conflict of interest.

## Publisher’s note

All claims expressed in this article are solely those of the authors and do not necessarily represent those of their affiliated organizations, or those of the publisher, the editors and the reviewers. Any product that may be evaluated in this article, or claim that may be made by its manufacturer, is not guaranteed or endorsed by the publisher.

## References

[1] Connell PP Hellman S . Advances in radiotherapy and implications for the next century: A historical perspective. Cancer Res (2009) 69(2):383–92. doi: 10.1158/0008-5472.CAN-07-6871 19147546

[2] Britel M Bourguignon M Foray N . The use of the term ‘radiosensitivity’ through history of radiation: From clarity to confusion. Int J Radiat Biol (2018) 94:503–12. doi: 10.1080/09553002.2018.1450535 29533136

[3] Foray N Bourguignon M Hamada N . Individual response to ionizing radiation. Mutat Res Reviews Mutat Res (2016) 770:369–86. doi: 10.1016/j.mrrev.2016.09.001 27919342

[4] Sato K Shimokawa T Imai T . Difference in acquired radioresistance induction between repeated photon and particle irradiation. Front Oncol (2019) 9:1213. doi: 10.3389/fonc.2019.01213 31799186 PMC6863406

[5] Alamilla-Presuel JC Burgos-Molina AM González-Vidal A Sendra-Portero F Ruiz-Gómez MJ . Factors and molecular mechanisms of radiation resistance in cancer cells. Int J Radiat Biol (2022) 98(8):1301–15. doi: 10.1080/09553002.2022.2047825 35225732

[6] Olivares-Urbano MA Grinan-Lison C Marchal JA Maria IN . CSC radioresistance: A therapeutic challenge to improve radiotherapy effectiveness in cancer. Cells (2020) 9:1651–85. doi: 10.3390/cells9071651 32660072 PMC7407195

